# Results of a prospective multicenter neuroendocrine tumor registry reporting on clinicopathologic characteristics of Greek patients

**DOI:** 10.1186/s12902-016-0089-7

**Published:** 2016-02-12

**Authors:** George C. Nikou, Kalliopi Pazaitou-Panayiotou, Dimitrios Dimitroulopoulos, Georgios Alexandrakis, Pavlos Papakostas, Michalis Vaslamatzis, Philippos Kaldrymidis, Vyron Markussis, Anna Koumarianou, on behalf of the “G-NET-Registry” investigators

**Affiliations:** Neuroendocrinology Section, 1st Department of Propaedeutic Internal Medicine, Laiko University Hospital, Athens, Greece; Unit of Endocrinology and Endocrine Oncology, Theagenio Cancer Hospital, Thessaloniki, Greece; Gastroenterology Department, Agios Savas Cancer Hospital, Αthens, Greece; Gastroenterology Department, ΝΙΜΤS Hospital, Αthens, Greece; Oncology Department, Hippokrateion Hospital, Athens, Greece; Oncology Department, Evangelismos Hospital, Athens, Greece; Department of Endocrinology, Metaxa Cancer Hospital, Piraeus, Greece; Ipsen epe, Athens, Greece; Hematology-Oncology Unit, Fourth Department of Internal Medicine, Attikon University Hospital, Medical School, National and Kapodestrian University of Athens, Rimini 1, 12462, Haidari, Athens, Greece

**Keywords:** Neuroendocrine tumors, NET, Neuroendocrine neoplasms, NEN, Neuroendocrine carcinomas, NEC, Gastric, Enteric, Pancreatic, Head and neck, Imaging, Therapy, Registry

## Abstract

**Background:**

The rare incidence of neuroendocrine neoplasms (NENs) has contributed to a paucity of large epidemiologic studies of patients with this condition. We investigated the occurrence and clinicopathologic features of NENs in Greece.

**Methods:**

Between October 2010 and November 2012 we collected data on 246 newly diagnosed patients from a broad-based multi-institutional registry that comprises eight academic and hospital sites in Greece. The WHO 2010 pathologic classification and the 7th AJCC Staging system was applied in all cases.

**Results:**

Of all patients 94 % had a sporadic and 6 % a multiple endocrine neoplasia tumor; 63.4 % were gastroenteropancreatic-(GEP)-NENs, 17.9 % Head & Neck NENs, 9.8 % NENs of Unknown Primary, 6.5 % Lung NENs and 2.4 % Pheochromocytomas. Gastric and pancreatic NENs were the most common primary sites. Poorly differentiated neuroendocrine carcinomas (NEC) were 9.3 %, all sporadic. Fifteen percent of patients were asymptomatic at presentation, 24 % had a first symptom of the disease related to endocrine syndrome and 61 % had symptoms related to locally advanced or metastatic disease. Metastatic disease was established in 25 % of tumors most frequently in the GEP NEN group. Findings are presented according to Ki-67 distribution. MRI had a higher diagnostic positive yield than Octreoscan. Somatostatin analogs, lanreotide and octreotide acetate, were prescribed at 38.5 & 61.5 % of NEN patients respectively and were found to be equally effective at providing symptomatic relief.

**Conclusions:**

This is to our knowledge the first study of a Greek tumor registry and one of the few European Registries providing information regarding clinicopathologic characteristics and therapies in patients with neuroendocrine tumors of various origin sites, beyond GEP NENs.

## Background

Neuroendocrine neoplasms (NENs) are a heterogeneous group of tumors arising from cells of the diffuse body neuroendocrine system most commonly in the gastrointestinal tract.

Since 1907, when originally described as benign tumors by Oberndorfer [1], the nomenclature and classification of NENs has changed several times, making the collection of epidemiological information and the comparison among studies published in the literature very difficult. Gastroenteropancreatic NENs (GEP NENs) were recently redefined according to the updated pathological and immunohistochemical criteria established by WHO [2]. Irrespective of the specific site of origin, GEP NENs are classified as ‘well differentiated’, ‘moderately differentiated’ or ‘poorly differentiated neuroendocrine carcinomas GEP-NEC’. Additionally the classification is based on the Ki-67 labeling index (LI) that categorizes GEP NENs into the three groups: ≤ 2, 3–20 and >20 % [2].

Despite a small number of national multicentric registry studies and an even smaller number of epidemiological studies the real incidence of NENs is unclear. This can be a reason that explains the discrepancy between the estimated incidence that differs between genders, races and among countries and continents [3, 4]. There are several explanations for this phenomenon most importantly that previous histopathologic classifications have been substituted by the most recent that encompasses more entities with malignant potential under the term neuroendocrine neoplasia [2, 5, 6].

Although there are several registries reporting on the incidence of GEP NENs [7–9] only few registries include data on NENs of other sites [10–13]. Therefore there is lack of information on the incidence and the relative frequency of many NEN subgroups including the head and neck and unknown primary (UP) that remain highly under-represented in the registries.

The primary endpoint of the study was to describe NEN subtypes in Greece and to collect information on the presenting symptoms, diagnostic evaluation, staging techniques and choice of treatment modalities, in these patients.

## Methods

### The neuroendocrine tumor registry in Greece

The Neuroendocrine Tumor Registry was generated by dedicated specialists involved in the diagnosis and treatment of NEN patients. The team included medical oncologists, endocrinologists and gastroenterologists from eight academic and non-academic tertiary hospitals with an established experience in the management of NEN patients. The registry was funded by IPSEN Epe, Greece. IPSEN had no influence on the setup of the database, data acquisition or data analysis and had no access to raw data. The design of the study was descriptive, multicenter and observational open-ended surveillance registry. Approval of the study was obtained from each Institutional Review Board & Ethics Committee according to the Declaration of Helsinki and Good Clinical Practice, as well as to the EU regulations [directives 95/46/EC (24/10/1995),2001/20/EC (04/04/2001) and (EC) 45/2001]. Regulatory approval was obtained from the National Organization for Medicines (EOF). All patients signed a written informed consent for participation in the study. No children were involved in this database.

### Data acquisition

A dedicated database software (TMS, Athens, Greece) was built with contribution by all authors during three specifically dedicated days. The items to be included were decided upon by specialists (medical oncologists, gastroenterologists and endocrinologists) experienced in the care of patients with NEN. This prospective survey was conducted in eight Greek referral centers and recruitment of NEN patients was started after October 2010. The feasibility and utility of the database was tested in a pre-test platform and necessary modifications were performed. The resulting database consisted of data fields divided in 18 sections including demographics, symptoms, tumor characteristics, diagnostic procedures, treatments modalities and outcome. If a patient received a treatment (e.g. somatostatin analogues) for more than one continuous treatment period, then the separate treatment periods and the response to each of them were documented as separate outcomes. Possible answers were either split into “yes” or “no” or selected from a drop-down menu. All physicians were trained and were responsible for the introduction of the studied features in the registry. One trained study monitor visited each center, reviewed the patients’ medical files provided by the institution and assessed the quality of data insertion to the database.

All patients were entered in the database, by trained doctors, with their initials, date of birth and date of histopathology diagnosis to exclude the possibility of more than one recordings of the same patient. Once the duplicates were removed this information was excluded. In the case of genetic syndrome data acquisition depended on its positive documentation of items. If for example multiple endocrine neoplasia (MEN) was documented either as a report of the genetic analysis or stated as diagnosis by the physician, the patient was documented as ‘MEN-positive’. In the database inserted parameters included also details on the diagnostic procedures and therapies applied. Patient data were specifically checked to avoid double insertions from different centers and in that case data were merged.

### Patient inclusion

Inclusion criteria were diagnosis of NEN histologically confirmed after October 2010 and a patient’s signed informed consent. Patients were excluded if they had a small or large cell lung cancer histology or if they were not actively followed up. Histological classification applied was the WHO 2010 classification [2] and staging was assessed according to the 7th AJCC Staging system [14].

### Statistical analysis

We performed an interim analyses after two years of patient recruitment in the registry. Continuous variables were expressed either with the use of the mean and the standard deviation or with the median and the minimum and maximum values depending on their distribution. The normality of the distribution of values was examined with the use of the Shapiro-Wilk test. Categorical variables were expressed as percentages (%). Statistical comparison between groups was performed with the use of Student’s t test or with the Mann Whitney U test when the distribution of variables was normal or not, respectively. Distribution of continuous variables between more than two groups was tested with the use of ANOVA or Kruskal-Wallis test, depending on the normality or not of their distribution. Differences in categorical variables were tested with the use of the Fisher’s Exact test. All statistical analyses were performed with the use of the IBM SPSS Statistics (ver. 22.0). All statistical tests were two-sided and significance was a priori determined at the *p* = 0.05 level.

## Results

### Patient population

During the study period, 246 eligible patients were recorded in the Greek NET Registry. Of these, 121 (49.2 %) were males. All patients were of Caucasian origin, except one of South Asian origin. The median age at diagnosis of all patients was 57 years (range 18–82).

Of all patients, 111 (45 %) were referred to the Registry Centers by a physician of a different specialty. Seventy six patients (31 %) were found incidentally during a diagnostic procedure for an apparently unrelated cause. One hundred fifty six NENs (63.4 %) were located in the gastroenteropancreatic system. The clinicopathologic characteristics are shown in Table 1. The more frequent localization in the stomach was the corpus, while it was the head for the pancreas. In the lung the typical NENs prevailed. The majority of tumors were well differentiated and with Ki-67 ≤ 2.

**Table 1:** Clinicopathologic characteristics of the study population

	*N* (%)
Primary site	
Gastrenteropancreatic	156 (63.4)
Stomach	54 (35 %)
Corpus	37 (68.5)
Antrum	11 (20.4)
Fundus	6 (11.1)
Pancreas	36 (23 %)
Head	20 (55.6)
Tail	10 (27.8)
Body	6 (16.7)
Duodenum	8 (5 %)
Jejunum	5 (3 %)
Ileum	10 (6 %)
Appendix	17 (11 %)
Colon	8 (5 %)
Rectum	18 (12 %)
Head and Neck^c^	44 (17.9)
Unknown Primary	24 (9.8)
Lung	16 (6.5)
Typical	10 (62.5)
Atypical	6 (37.5)
Pheochromocytoma	6 (2.4)
Differentiation Grade^a^	
Well	132 (58.4)
Moderate	73 (32.3)
Poor	21 (9.3)
Ki-67 ^b^	
≤2 %	166 (72.8)
3–20 %	56 (24.5)
>20 %	6 (2.6)

^a^20 missing cases

^b^18 missing cases

^c^includes 41 MTC and 3 paragangliomas

Of the 228 evaluable patients, 6.5 % had T4 stage (all GEP NENs), 28 % had lymph nodal infiltration (most commonly GEP NEN, bronchial and H&N) and 24 % had metastatic disease most frequently in the UP and GEP NEN groups. Data regarding the T stage are missing for 18 UP patients.

Multiple endocrine neoplasia was diagnosed in 14 patients (6 %), all MEN1. Mean age at diagnosis in patients with MEN1 syndrome was 48.8 ± 19 years compared to 56 ± 14 years in non-MEN patients (*p* = 0.131). Eight (57 %) had parathyroid hyperplasia, 3 (21 %) pituitary adenoma and 4 (29 %) adrenal adenomas. Neuroendocrine tumors associated with MEN1 syndrome involved 4 head and neck, 1 bronchial, 4 GEP, 2 pheochromocytomas and 3 unknown primary (UP). Clinical syndrome due to hormone secretion was present in 7 patients.

### Clinical manifestations of NEN

Of all patients 38 patients (15 %) were asymptomatic, 24 % reported symptoms related to endocrine syndrome and 61 % had symptoms related to the presence of the tumoral mass. The most common clinical symptoms related to advance disease were dyspepsia (44 %), abdominal pain (38.6 %) and asthenia (35.8 %) followed by weight loss (18.3 %) and anorexia (16.7 %). Symptoms related to hormonal secretion were less reported and included mainly flushing (14.5 %), diarrhea (12 %) or both (1 %).

Regarding the symptoms according to tumor location, in gastroenteric NENs comprised abdominal pain (41 %), asthenia (41 %), weight loss (35 %), dyspepsia (35 %), and anorexia (17 %). Pancreatic NENs presented most commonly with abdominal pain (52 %), diarrhea (28 %), weight loss (28 %), dyspepsia (26 %), and asthenia (21 %). For head and neck NENs (medullary thyroid carcinomas and paragangliomas) the most common presentation was neck swelling (14 %). For adrenal NENs the most common manifestations were asthenia (25 %), weight loss (12.5 %), hyperglycemia (12.5 %), and sweating (25 %). Vein thrombosis was reported in head and neck NENs (2.5 %) but not in GEP NENs.

### Diagnostic procedures

The diagnostic procedures applied according to NEN site of origin are shown in Table 2. Most commonly performed test for staging was computed tomography (CT; 82 % of patients), followed by Octreoscan (63.4 %). The procedures leading to the highest percentage of positive results (% of positive results with imaging compared to positive histopathological results) were 2-deoxy-2-(^18^F) fluoro-D-glucose (^18^F-FDG) positron emission tomography (PET)-computed tomography (CT) scan and magnetic resonance.

**Table 2:** Diagnostic procedures used for staging, according to primary site (absolute number performed and % positive yield)

Diagnostic Procedure	Head &Neck^a^	Bronchial	GEP	Pheochromocytoma	Unknown Primary	Total
	*N* = 44	*N* = 16	*N* = 156	*N* = 6	*N* = 24	*N* = 246
(18) F-FDG PET/CT	6 (100.0)	1 (100.0)	3 (66.7)	0 (0.0)	1 (0.0)	11 (81.8)
Magnetic Resonance	12 (81.8)	4 (100.0)	52 (69.2)	3 (100.0)	14 (78.6)	85 (75.0)
Endoscopic Ultrasound	1 (0.0)	0 (0.0)	21 (81.0)	0 (0.0)	5 (60.0)	27 (74.1)
Bronchoscopy	0 (0.0)	7 (100.0)	0 (0.0)	0 (0.0)	3 (0.0)	10 (70.0)
MIBG	1 (0.0)	0 (0.0)	1 (100.0)	5 (80.0)	3 (33.3)	10 (60.0)
Enteroclysis	0 (0.0)	0 (0.0)	4 (75.0)	0 (0.0)	1 (0.0)	5 (60.0)
Computed Tomography	33 (78.8)	16 (93.8)	126 (43.2)	4 (100.0)	23 (81.8)	202 (58.5)
Abdominal Ultrasound	5 (0.0)	3 (33.3)	65 (50.8)	4 (100.0)	13 (92.3)	90 (55.6)
Gastroscopy	4 (0.0)	3 (0.0)	105 (60.0)	0 (0.0)	14 (14.3)	126 (51.6)
Octreoscan	16 (68.8)	11 (54.5)	107 (44.9)	2 (50.0)	20 (65.0)	156 (50.6)
Colonoscopy	2 (0.0)	2 (0.0)	71 (38.0)	0 (0.0)	15 (0.0)	90 (30.0)
Bone Scan	22 (18.2)	12 (25.0)	10 (22.2)	4 (25.0)	11 (18.2)	59 (20.7)
Endoscopic Capsule	0 (0.0)	0 (0.0)	10 (30.0)	0 (0.0)	6 (0.0)	16 (18.8)
x-Ray	33 (0.0)	15 (100.0)	85 (2.4)	2 (0.0)	17 (17.6)	152 (13.2)

2-deoxy-2-(^18^F) fluoro-D-glucose: positron emission tomography/computed tomography (18F-FDG PET/CT), n: number of patients

^a^includes 41 MTC and 3 paragangliomas

Investigation of biochemical markers were carried out for serum chromogranin A (CgA) and for neuron specific enolase (NSE) in the serum of 60 and 43 % of patients respectively and by urinary 5-HIAA in 38 % of patients. The positive yield of these tests were 89, 28 and 33 % respectively, most commonly all three positive in NEN of UP. In patients with a suspected functioning NEN additional hormonal assessment included insulin (6.5 %), glucagon (4.1 %), gastrin (40.7 %) and VIP (3.3 %).

### Histopathological characteristics

The histopathological features of all NEN patients are shown in Table 1. Specific characteristics such as vascular invasion, local infiltration and differentiation grade in relation to primary site are presented in Table 3. Local infiltration was present in 55 (38.7 %) and vascular invasion in 23 (16.7 %) of the 156 patients with GEP NENs. Ki-67 labeling index (LI) was carried out in 228 (93 %) NENs. The distribution of Ki-67 according to the primary site is shown in Table 3 and according to gender and staging in Table 4. Immunohistochemical staining for serum CgA, synaptophysin and somatostatin receptors (SSTR-2) was done in 186 (76 %), 162 (66 %) and 43 (17 %) of the entire cohort and was positive in 165 (89 %), 147 (91 %) and 31 (72 %) of the tested patients respectively.

**Table 3:** Distribution of vascular invasion, local infiltration, differentiation grading and Ki-67 LI in relation to primary site (absolute number and % of patients)

Clinicopathologic features	Head &Neck^a^	Bronchial	GEP	Pheochromocytoma	Unknown Primary	Total
	*N* = 44	*N* = 16	*N* = 156	*N* = 6	*N* = 24	
Vascular invasion	13 (28.2)	5 (10.8)	23 (50.0)	2 (4.3)	3 (6.5)	46 (100.0)
Local infiltration	22 (24.4)	6 (6.6)	55 (61.1)	2 (2.2)	5 (5.5)	90 (100.0)
Differentiation grading						
G1	22 (16.6)	10 (7.5)	84 (63.6)	4 (3.0)	12 (9.0)	132 (100.0)
G2	18 (24.6)	6 (8.2)	42 (57.5)	2 (2.7)	5 (6.8)	73 (100.0)
G3	1 (4.7)	0 (0)	17 (80.9)	0 (0)	3 (14.2)	21 (100.0)
Ki-67 labelling index						
Ki-67 ≤ 2	34 (20.4)	13 (7.8)	104 (62.6)	5 (3.0)	10 (6.0)	166 (100.0)
Ki-67 3-20	3 (5.3)	3 (5.3)	39 (69.6)	1 (1.7)	10 (17.8)	56 (100.0)
Ki-67 > 20	0 (0)	0 (0)	3 (50.0)	0 (0)	3 (50.0)	6 (100.0)

^a^includes 41 MTC and 3 paragangliomas

**Table 4:** Distribution of Ki-67 labelling index according to gender, stage, type of treatment

Parameters	Ki-67 Labeling Index, *n* (%)			Total *n*
	≤2 (166 cases)	3–20 (56 cases)	>20 (6 cases)	
Gender				
Male	82 (74.5)	24 (21.8)	4 (3.6)	110 (100.0)
Female	84 (71.2)	32 (27.1)	2 (1.7)	118 (100.0)
Stage TNM				
Τ0	16 (84.2)	3 (15.8)	0 (0)	19 (100.0)
Τ1	68 (82.9)	14 (17.1)	0 (0)	82 (100.0)
Τ2	16 (64.0)	9 (36.0)	0 (0)	25 (100.0)
Τ3	17 (54.8)	11 (35.5)	3 (9.7)	31 (100.0)
Τ4	4 (28.6)	10 (71.4)	0 (0)	14 (100.0)
ΤΧ	45 (78.9)	9 (15.8)	3 (5.3)	57 (100.0)
Ν0	111 (78.2)	30 (21.1)	1 (0.7)	142 (100.0)
Ν1	39 (60.9)	20 (31.3)	5 (7.8)	64 (100.0)
ΝΧ	16 (72.7)	6 (27.3)	0 (0)	22 (100.0)
Μ0	97 (81.5)	22 (18.5)	0 (0)	119 (100.0)
Μ1	29 (52.7)	21 (38.2)	5 (9.1)	55 (100.0)
ΜΧ	40 (74.1)	13 (24.1)	1 (1.8)	54 (100.0)
Type of treatment				
Lanreotide	52 (69.3)	20 (26.6)	3 (4.1)	75 (100.0)
Octreotide	85 (71.4)	33 (27.7)	1 (0.9)	119 (100.0)
Sunitinib	5 (50.0)	5 (50.0)	0 (0)	10 (100.0)
Everolimus	3 (60.0)	2 (40.0)	0 (0)	5 (100.0)
Chemotherapy	11 (50)	9 (40.9)	2 (9.1)	22 (100.0)

### Therapeutic interventions

Treatments applied in NEN patients are shown in Tables 4, 5 and 6.

**Table 5:** Primary surgery performed in NEN patients (*n*)

Primary Site (all Ki-67 < 20 %)	Radical Surgery (*n*)	Non Radical Surgery (*n*)	Total
Head and Neck	40	4	44
Lung	8	2	10
GEP	110	20	130
Pheochromocytoma	4	2	6
Unknown Primary	2	1	3
Type of Surgery	(n)		
Open Surgery	128		
Endoscopic/Laparoscopic Excision	65		

*GEP* gastroenteropancreatic, *n* number of patients

**Table 6:** Surgery and debulking procedures performed in NEN patients (n) with liver and lung metastases

	Number of patients	Primary Site (all Ki-67 < 20 %)
Patients with Liver Metastases	Total number: 49	34 GEP, 11 UP, 3 lung, 1 pheochromocyroma
Liver Metastasectomy	6	4 GEP, 1 Lung, 1 unknown primary
Chemoembolization	3	GEP
Radiofrequency Ablation	4	GEP
Other	2	GEP
Patients with Lung Metastases	Total number: 6	4 GEP, 1 lung, 1 unknown primary
Surgical Excision	1	GEP

*GEP* gastroenteropancreatic, *UP* unknown primary

Somatostatin analogs were administered in 135 patients. Lanreotide 60–120 mg and Octreotide 20–30 mg LAR was delivered for a total number of 1378 and 2473 months, respectively. Improvement of symptoms was observed in 68.2 %, stabilization in 25.9 % and deterioration in 5.8 % of the patients. There was no difference between the symptom responses observed with the two analogues (*p* = 0.295), even after controlling for the different distribution in Ki-67 LI categories in a multinomial logistic regression.

Chemotherapy was given in 22 patients and targeted therapies in 15 patients. Interferon was recorded as treatment in only 2 patients. Bevacizumab, an anti-VEGF monoclonal antibody, was delivered in 4 GEP NEN patients. Peptide Receptor Radionuclide Therapy was delivered in 17 patients and radiotherapy in 9 patients. Surgical excision was applied in 193 patients with complete excision achieved in 164 patients, all with Ki-67 < 20 % (Table 5). Local therapies in patients with liver metastases were applied on 15 occasions (Table 6).

### Deaths observed

During the follow-up period of the registry and at the time of the analyses 10 deaths were documented, corresponding to 4.1 % of the registry population. The primary sites of origin of these patients were 6 gastroenteric, 2 pancreatic and 2 pheochromocytomas.

## Discussion

This study presents for the first time the data of the NET registry in Greece focusing on the epidemiologic and clinico-pathologic characteristics as well as the therapeutic modalities applied in patients with all type of NENs except small and large cell lung cancer.

Greece is a European country with a reported population of 11 million in 2012 (http://countryeconomy.com/demography/population/greece). This partly explains the small number of recorded patients (246) presented in the participating centers during the first two years of this observational study. Taken into consideration that the eight centers participating in the study do not cover for the entire population definitive conclusions on the incidence of NEN tumors cannot be made.

Only few cancer NEN registries exist in the USA and Europe, mostly national. According to the SEER database (seer.cancer.gov website), that includes information on 7,262,696 cancer patients, covering for 28 % of the USA population the incidence of NENs in 2004 is 5.25/100.000 inhabitants [15]. On the other hand, data on the incidence of NENs in Europe, is limited and is usually reported by anatomic location, most commonly GEP NENs [8, 16]. Specifically, in one study including NENs of all sites, except lung, conducted by the RareCare Working Group, the overall incidence rate was 25/1,000,000 in total but it was highest when patients older than 65 years of age were considered (40 per 1,000,000) [17].

In our registry, the median age at diagnosis and the gender’s ratio were in accordance to those reported in other published registries [4, 10, 12]. We found the gastroenteropancreatic tract being the most common followed by the head and neck and UP. This is slightly different from that reported in one study from the Mediterranean area with pancreas and lung being the commonest primaries, where it was found that 63 % were GEP-NENs, 33 % thoracic-NENs including thymic, 4 % UP-NEN [10]. This difference is probably due to the fact that there was no center for lung NENs participating in our registry. With respect to the GI tract we found that gastric (35 %), pancreatic (23 %) and rectal NENs (12 %) were the commonest primary sites. An inverse frequency was found in the Italian study as the pancreatic primary (31 %) was commonest compared to gastric (10 %) [10].

The presenting symptoms of our patients were related to endocrine syndrome in 24 %, and to mass effect in 61 %, most commonly dyspepsia, similarly to our previous retrospective study [18]. Although only 15 % of patients were completely asymptomatic, the diagnosis was considered ‘incidental’ in 30.9 % as many patients had vague and under-reported symptoms recognized after the diagnosis. In line with our study a registry from Italy reported that the first symptoms of the disease were related to tumor burden in 46 %, endocrine syndrome in 23 %, while the diagnosis was fortuity in 29 % of cases [10].

Six percent of our patients had MEN syndrome diagnosed earlier compared to patients with sporadic NENs. Multiple endocrine neoplasia associated NEN frequency was not different from that reported in an Italian (7 %), a Spanish (5 %) and a Japanese registry (4.3 %) [8, 10, 19]. As for other tumors associated with hereditary syndromes, NENs associated with MEN mandates genetic screening of the relatives, different surveillance methods and treatments for the involved patients [20, 21].

The differentiation grade and Ki-67 LI are not only obligatory requirements of the pathological classification system but represent the upmost important prognostic factors and may help tailor treatment in NEN patients [22]. Descriptions regarding the Ki-67 LI and differentiation grade are lacking in the published NEN registries and this is due not only to the frequent change in the classification system but also to the underreporting of the Ki-67 in the histological diagnosis [13]. For 93 % of our histological specimens Ki-67 LI was counted and found most commonly <2 %. With respect to the differentiation grade we could find only one similar study that reported a 52 % of well differentiated neuroendocrine tumors (NETs) and a 13 % of poorly differentiated NEC [10].

Head and neck was the second most common (20 %) primary site in our registry and included medullary tumors and paragangliomas. To our knowledge there is no information from other registries on these NENs and thus we cannot have comparable data. One possible explanation is that only in the past few years medullary tumors were included in the large family of NENs.

NENs of UP was the third most frequent subtype (9.8 %) in our registry, most commonly of Ki-67 LI > 2 % and G2/G3. A European study reported a lower incidence (4 %) and a USA study 13 % of UP [10, 15]. By definition NEN of UP site refers to a group of patients with locally advanced disease such as regional lymphadenopathy or most commonly metastatic disease [23]. It cannot be identified if these tumors arise from an occult gastrointestinal or pulmonary primary site or else from a multipotent stem cell [24]. One explanation for this high frequency of UP is the non-availability in our country of Gallium-68 PET/CT and DOPA PET/CT that have a higher sensitivity for the identification of NEN primaries such as GEPs and medullary/paragangliomas respectively [25, 26]. A recent systematic review of the studies published in the literature did not identify differences in the biology of UP NENs or in the outcome of these patients compared to NEN patients with known primary matched for grade [27].

In our study we found metastatic disease at presentation in 25 % of patients comprising 17.5 % of well, 37.5 % of moderately and 83 % of poorly differentiated neoplasms. Similar findings were reported from the SEER database as distant metastases at diagnosis comprised 21 % well, 30 % moderately and 50 % poorly differentiated tumors [15].

In our study we did not find a statistically significant difference in the distribution of histologies, primaries and staging between males and females. However, in the SEER database male patients were more likely to have metastasis at presentation, than female patients, in a statistically significant way [15]. We were able to detect in our tumors local and vascular infiltration two well-established features of malignant behavior with adverse prognostic significance [5]. As long term data of our cohort is not yet matured we cannot comment on the impact of these features on the survival of our patients.

With respect to diagnostic imaging, in our population imaging with internationally recommended techniques such as somatostatin receptor PET/CT imaging with Gallium-68 and endoscopic ultrasound (EUS) is either unavailable (^68^Ga) or accessible in very few sites (EUS). In our study octreoscan was less sensitive compared to other procedures such as computed tomography. This is in line with an earlier study by our group showing that octreoscan compared to conventional imaging such as ultrasound and CT is less sensitive for the detection of liver metastases [28].

The most commonly applied therapy in our cohort was somatostatin analogs (SSA), octreotide 30 mg LAR and lanreotide 120 mg Autogel, both with an established role in the symptom and tumor control of patients with well and moderately differentiated NENs [29–31]. Recently, in the CLARINET trial, lanreotide 120 mg Autogel was established for its anti tumoral effect in both pancreatic and gastroenteric locally advanced or metastatic neuroendocrine tumors with Ki-67 up to 10 %. In this registry, the response was documented as improvement, stabilization or deterioration of the symptoms, without the use of the RECIST criteria, since this was out of the scope of the registry. Similarly to the previously published studies, our findings indicate an equal role of the different SSAs in the control of NEN symptoms.

Other therapies applied in our patients with Ki-67 LI < 20 % included systemic chemotherapy, with either streptozocin/5FU or temozolomide, or a targeted agent such as everolimus and sunitinib, according to previously published data [32–35]. Patients with poorly differentiated tumors or Ki-67 > 20 % were treated with systemic chemotherapy comprising cisplatin/carboplatin and etoposide doublets according to established evidence [36].

Limitations of our study include a) the fact that not all NENs diagnosed in our country between October 2010 and November 2012 were included in the present registry and b) the lack of information on survival, progressive free survival, recurrences and new metastases. As long term data of our cohort is not yet matured we cannot comment on the impact of our findings on the evolution of the disease in general and on overall survival.

## Conclusions

We present for the first time the results of a Greek NET registry that includes NEN from a variety of primary sites. Our results indicate some differences in the occurrence of NENs reported from registries of other European countries and the USA. It is thus important to develop national registries for the precise description of the incidence and the handling of NENs and the possible application of the findings in prevention and pharmacoeconomics. Based on the reported occurrence of NENs and the variations in incidence observed in the literature it is important that each country develops a national registry for the recording of the incidence and clinico-pathologic characteristics of these rare tumors and to improve our understanding of the biology and survival of these tumors.
